# Interference-Aware Routing for Difficult Wireless Sensor Network Environment with SWIPT

**DOI:** 10.3390/s19183978

**Published:** 2019-09-14

**Authors:** Shiming He, Yangning Tang, Zhuozhou Li, Feng Li, Kun Xie, Hye-jin Kim, Gwang-jun Kim

**Affiliations:** 1 School of Computer and Communication Engineering, Hunan Provincial Key Laboratory of Intelligent Processing of Big Data on Transportation, Changsha University of Science and Technology, Changsha 410114, China; smhe_cs@csust.edu.cn (S.H.); tee@stu.csust.edu.cn (Y.T.); lee@stu.csust.edu.cn (Z.L.); lif@csust.edu.cn (F.L.); 2 Key Lab of Broadband Wireless Communication and Sensor Network Technology (Nanjing University of Posts and Telecommunications), Ministry of Education, Nanjing 210003, China; 3 College of Computer Science and Electronics Engineering, Hunan University, Changsha 410082, China; cskxie@gmail.com; 4 Leader of Research Management Team, Academic Times, Seoul 136170, Korea; 5 Department of Computer Engineering, Chonnam National University, Gwangju 61186, Korea

**Keywords:** harsh environment, wireless sensor network, simultaneous wireless information and power transfer, interference, route

## Abstract

The main challenges of sensing in harsh industrial and biological environments are the limited energy of sensor nodes and the difficulty of charging sensor nodes. Simultaneous wireless information and power transfer (SWIPT) is a non-invasive option to replenish energy. SWIPT harvests energy and decodes information from the same RF signal, which is influencing the design of a wireless sensor network. In multi-hop multi-flow wireless sensor networks, interference generally exists, and the interference has a different influence on SWIPT. Route, interference and SWIPT are dependent. However, existing works consider SWIPT link resource allocation with a given route or only select path for one flow without interference. Therefore, this paper firstly analyzes the influence of interference on SWIPT, and select the SWIPT routing with interference. We design an interference-based information and energy allocation model to maximize the link capacity with SWIPT. Then, we design an interference-aware route metric, formulate SWIPT routing problem, and design an interference-aware SWIPT routing algorithm. The simulation results show that as the number of flows increases, there is more likely to obtain performance gains from interference and SWIPT.

## 1. Introduction

Sensing in harsh industrial and biological environments, such as the human body and concrete, has attracted a lot of recent interests. However, the main challenges of sensing in harsh industrial and biological environments are the limited energy of sensor nodes and the difficulty of charging sensor nodes. After sensors being deployed, it is impossible to change them or charge them by solar or wind because of the closed environment except invading the human body or breaking the concrete. Therefore, non-invasive energy replenishment is the best choice for these difficult environments. Simultaneous wireless information and power transfer (SWIPT) [1] is a non-invasive option to replenish energy. With the help of SWIPT, wireless sensor networks (WSNs) [2,3,4] can prolong the life of sensor nodes without invasion.

SWIPT is a kind of energy harvesting mechanism based on radio frequency (RF) in which the receiver captures the ambient RF radiation and converts it into a direct current voltage through rectennas circuits [5]. Not only the ambient RF radiation but also the desired information signals are harvested by the receiver of SWIPT. That is, the same RF signal received by receiver supports to harvest energy and decode information at the same time by special information energy splitting mechanisms. Compared with other energy sources, such as solar energy and wind energy, the desired RF is more reliable. SWIPT provides a new alternative for energy-constrained wireless devices to harvest energy and prolong the network lifetime. SWIPT is valuable in the future development of wireless sensor networks in harsh environments.

There are several advantages in wireless network with SWIPT: (1) SWIPT utilizes the desired radio frequency as energy source which is reliable and controlled; (2) sensors do not deploy the plugs for charging; (3) the sensors’ main functions, such as sensing, sending and receiving, can work as normal while SWIPT is used.

However, the system design of SWIPT is more complex to exploit those advantages. Several techniques and models have been proposed for the architecture design of receivers in the literature, e.g., [6,7,8]. Currently, SWIPT is applied in cellular networks [9,10] and two-hop cooperative networks [11,12] to improve the network performance.

After that, how to extend SWIPT to the multi-hop wireless network [13,14,15] has attracted interests [16,17]. Guo et al. [16] points that SWIPT can keep more remaining energy of nodes and balance the remaining energy among nodes. However, the interference [18] generally exists which is that one directional link affects another directional link [19,20] while the interference is that uplink affect the downlink performance in cellular system. The interference makes different influence on SWIPT compared to wireless information transmission (WIT) where RF signal is only decoded into information. Existing works consider SWIPT link resource allocation with a given route or only select path for one flow without interference.

For enhancing the performance of a difficult wireless sensor network with SWIPT, our work jointly takes account of interference, SWIPT and routing to design interference-aware SWIPT routing. However, applying SWIPT to interference environment of multi-hop multi-flow difficult wireless sensor network faces lots of challenges which are listed.

Firstly, the interference makes a different influence on SWIPT compared to WIT. On one hand, the interference decreases signal-to-interference-plus-noise ratio (SINR) and reduces the quality of information transmission. On the other hand, the interference improves the power of the received signal, and further increases the received energy. It means that interference becomes a source of energy. Interference is not negative for SWIPT. How to allocate information and energy depends on interference.

Secondly, the interference is produced by the routes of previous flows. How to allocate information and energy determines the links among nodes and network topology which decide the route. The links and network topology vary as the route selection of previous flows and allocation between information and energy. Route, interference and SWIPT are interactive.

Finally, two transmission modes, such as SWIPT and WIT, could be taken by nodes. Routing is more complicated. The performance of two transmission modes varies from interference. It is necessary to decide to transmit with WIT or SWITP and allocate information and energy carefully while finding the path in multi-hop multi-flow WSNs.

To sum up, routing, interference, and information and energy allocation are inter-dependent. To produce a marked effect of SWIPT technique on WSN with interference and multiple flows, the necessity of solving these problems together is recognized.

In this paper, we firstly analyze the influence of interference on SWIPT and solve the multiple flows routing problem with SWIPT according to the impact of interference. The contributions of our work are summarized.

- We firstly propose an interference-based information and energy allocation model to analyze the influence of interference on SWIPT and maximize the capacity of SWIPT link. This allocation model is solved by a Lagrange multiplier algorithm.
- We design a novel metric to evaluate the capacity of link and path under interference. With the benefit of the metric, we formulate the interference-aware SWIPT routing problem.
- An interference-aware SWIPT routing algorithm is proposed to calculate the interference from previous flows to get the maximum capacity while finding path. The maximum capacity is shared by the current flows. The route is selected by the shared maximum capacity.
- The performance of our proposed scheme is characterized and analyzed by simulations. The simulation results verify that the significant capacity gains by balancing SWIPT and interference in multi-hop multi-flow WSNs. As the number of flows increases, there is more likely to obtain performance gains from interference and SWIPT.

The remainder of this paper is organized as follows. Section 2 briefly reviews the related works. Section 3 includes the system models, motivation and solution overview. Section 4 proposes a maximum capacity allocation model to analyze the influence of interference on SWIPT. Section 5 formulates routing problem with SWIPT and interference and design algorithms. Section 6 uses simulations to analyze the performance. Section 7 concludes the work.

## 2. Related Work

Although there are several energy-efficient algorithms [21,22,23], the node’s energy will exhaust one day. Harvesting energy from environment is the best way to prolong the life of wireless nodes and provide the power to finish more complex data process task, i.e., AI task [24,25,26,27,28] in mobile or embedding device, except for data collection. For harvesting energy from the desired information signal, the architectures of SWIPT receiver have been designed [6,7,8], which are two practical information energy splitting mechanisms including time switching (TS) and power splitting (PS). The receiver with TS periodically switches to decode information and harvest energy, while the receiver with PS divides signal into two portions with separate power. One portion is used for information decoding, and the other is used for energy harvesting.

The research about SWIPT mainly focus on the cellular networks  [29,30,31,32,33,34,35,36,37] or cooperative networks [38,39,40,41,42,43,44,45,46]. Receiver with SWIPT should balance information decoding and energy harvesting for various performance goals in different contexts, i.e., less transmitting power, higher transmission rate, more received power, or better energy efficiency.

Researches are starting to introduce SWIPT into multi-hop network [47,48]. SWIPT is applied into a multi-hop mobile WSN [16] which shows that SWIPT can keep more remaining energy of nodes and balance the remaining energy among nodes. Ref. He et al. designs a SWIPT routing algorithm to select path with less energy consumption. However, interference is not taken account into in [16,17]. Ref. [16] allocates the SWIPT link resource with a given route selected by AODV, that is, the path selection and SWIPT link resource allocation are separated. Ref. [17] designs SWIPT routing for only single flow without interference.

## 3. System Model

In this section, network models, channel models, and receiver models are introduced, an example is presented to show our motivation. At last, an framework of our scheme is illustrated. Table 1 summarizes the notations and definitions used in this paper.

### 3.1. Network Models

**Figure 1:**
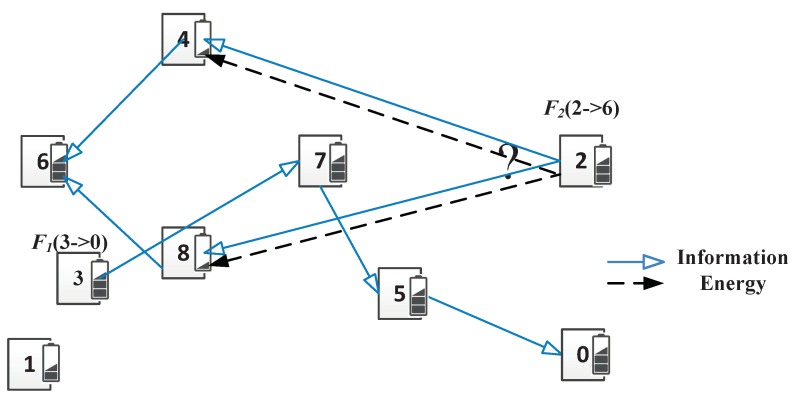
Network Model. There are multiple concurrent flows. There exists a flow $F_{1}\left(3\to 0\right)=3\to 7\to 5\to 0$. Which path is better for a new arrived flow $F_{2}\left(2\to 6\right)$, path 2→4→6 with less interference or path 2→8→6 with more interference?

A WSN with *N* nodes is shown in Figure 1. Both the sender and receiver are equipped with one antenna, while the case of the multiple antennas on each node will study in future work [49,50]. Each sensor node is deployed by battery and have uniform storage and computation capabilities. A node *i*’s residual energy is denoted by $Er_{i}$. When the node *i*’s residual energy $Er_{i}$ is not higher than $Er_{min}$, the node *i* does not forward packets for other nodes to avoid consuming its energy.

*M* concurrent flows exist, denoted by a set $F=\{F_{1},F_{2},\ldots ,F_{M}\}$. Source node and destination node of a flow $F_{i}\left(S_{i}\to D_{i}\right)$ are denoted by $S_{i}$ and $D_{i}$, respectively. The data from source to destination passes multiple nodes. For example, there is a flow $F_{1}\left(3\to 0\right)=3\to 7\to 5\to 0$. Which path is better for a new arrived flow $F_{2}\left(2\to 6\right)$, path 2→4→6 with less interference or path 2→8→6 with more interference?

### 3.2. Channel Models

At the sender side, the complex baseband signal is denoted by x(t). We assume that x(t) is a narrow-band signal with bandwidth of *B* Hz, and $E[|x\left(t\right){|}^{2}]=1$, where E[·] and |·| represent the statistical expectation and the absolute value, respectively. The wireless channel $h_{ij}$ is the channel from *i* to *j* with channel gain $|h_{ij}{|}^{2}$ which captures the effects of path-loss, shadowing, and fading.

**Figure 2:**
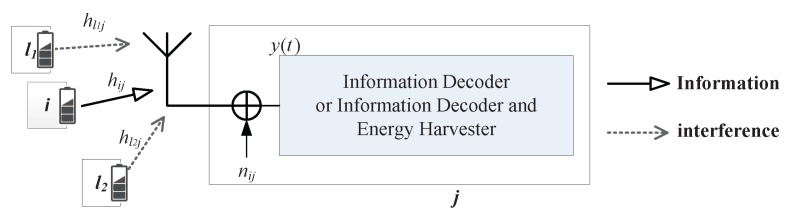
The channel model.

There is interference from other flows in multiple flows networks, as shown in Figure 2. $x_{l}\left(t\right)$ denotes the interference signal at the receiver from node *l* with power $P_{l}$, and $h_{lj}$ denotes the channel power gain between *l* and *j*. The received RF signal is y(t)

(1) $$y\left(t\right)=\sqrt{P_{ij}}h_{ij}x\left(t\right)+\sum_{l\in \Phi_{j}}\sqrt{P_{l}}h_{lj}x_{l}\left(t\right)+n_{ij}\left(t\right),$$

where $\Phi_{j}$ is the node set interfered with node *j*, $n_{ij}$ is the noise produced by antenna and $n_{ij}∼CN\left(0,\sigma_{ij}^{2}\right)$. $CN(\mu ,\sigma^{2})$ represents the circularly symmetric complex Gaussian (CSCG) distribution, μ is mean, $\sigma^{2}$ is variance, and ∼ represents “distributed as”.

### 3.3. Receiver Models

**Figure 3:**
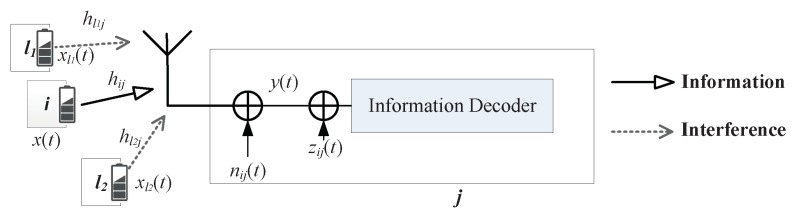
Wireless information transmission (WIT) receiver.

**Figure 4:**
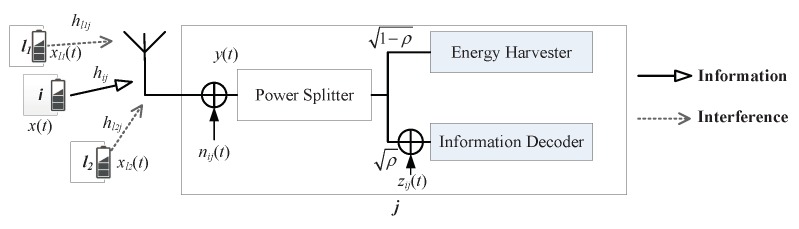
Simultaneous wireless information and power transfer (SWIPT) receiver.

There are two kinds of receivers, WIT receiver and SWIPT receiver, as shown in Figure 3 and Figure 4.

#### 3.3.1. WIT Receiver

WIT is the most common communication method in current wireless networks, where the RF signal received by the receive node is all used by the signal processing circuit for information decoding (ID).

The standard architecture of a WIT receiver is shown in Figure 3. The signal-to-interference- plus-noise ratio (SINR) on WIT receiver can be obtained by Equation (2).

(2) $$\gamma_{ij}^{IWIT}={\left|h_{ij}\right|}^{2}P_{ij}/\left(\sigma_{ij}^{2}+\eta_{ij}^{2}+\sum_{l\in \Phi_{j}}P_{l}{\left|h_{lj}\right|}^{2}\right),$$

where the noise produced by signal conversion is denoted by $z_{ij}$ with $z_{ij}∼CN\left(0,\eta_{ij}^{2}\right)$. The noise produced by signal conversion is introduced by the circuit of information decoding or signal conversion which do not exist in the circuit of energy harvesting. However, the noise produced by the antenna is common for information decoding and energy harvesting. Therefore, we separate the noise produced by the antenna from the noise produced by the signal conversion. The channel capacity at the receiver is

(3) $$C_{ij}^{WIT}=W*\log_{2}\left(1+\gamma_{ij}^{IWIT}\right),$$

where *W* denotes the fading channel bandwidth.

#### 3.3.2. SWIPT Receiver

The receiver node *j* still receives the same signal y(t) from the sender with interference. The power splitting (PS) mode of SWIPT receiver architecture [7] is taken into account in this paper. Therefore, the received RF signal is divided into two portions with a power splitting ratio $\rho_{ij}\in \left[0,1\right]$. One portion $\sqrt{1-\rho }y\left(t\right)$ is fed into the energy harvesting (EH) circuit.

(4) $$y^{EH}\left(t\right)=\sqrt{1-\rho_{ij}}y\left(t\right).$$

The harvested power is obtained by Equation (5) [8].

(5) $$E_{ij}^{eh}=\varepsilon \left(1-\rho_{ij}\right)\left(\right|h_{ij}{|}^{2}P_{ij}+\rho_{ij}\sigma_{ij}^{2}+\rho_{ij}\sum_{l\in \Phi_{j}}P_{l}{\left|h_{lj}\right|}^{2},)$$

where the energy converting coefficient of EH circuit is denoted by ε∈[0,1] (100×ε% of the received energy can be stored).

The other portion of RF signal $\sqrt{\rho }y\left(t\right)$ is used by the signal processing or information decoding circuit. The received signal for decoding information is given by Equation (6).

(6) $$y^{ID}\left(t\right)=\sqrt{\rho_{ij}}\left(y\left(t\right)+z_{ij}\left(t\right)\right).$$

The SINR at the SWIPT receiver is derived as

(7) $$\gamma_{ij}^{ISWIPT}=\rho_{ij}{\left|h_{ij}\right|}^{2}P_{ij}/\left(\rho_{ij}\sigma_{ij}^{2}+\eta_{ij}^{2}+\rho_{ij}\sum_{l\in \Phi_{j}}P_{l}{\left|h_{lj}\right|}^{2}.\right)$$

The capacity of link with SWIPT is given by Equation (8).

(8) $$C_{ij}^{SWIPT}=W*\log_{2}\left(1+\gamma_{ij}^{ISWIPT}\right)$$

If the power splitting ratio is 1, the SWIPT receiver is equal to a WIT receiver. Therefore, the SWIPT receiver can also use wireless information transmission.

**Figure 5:**
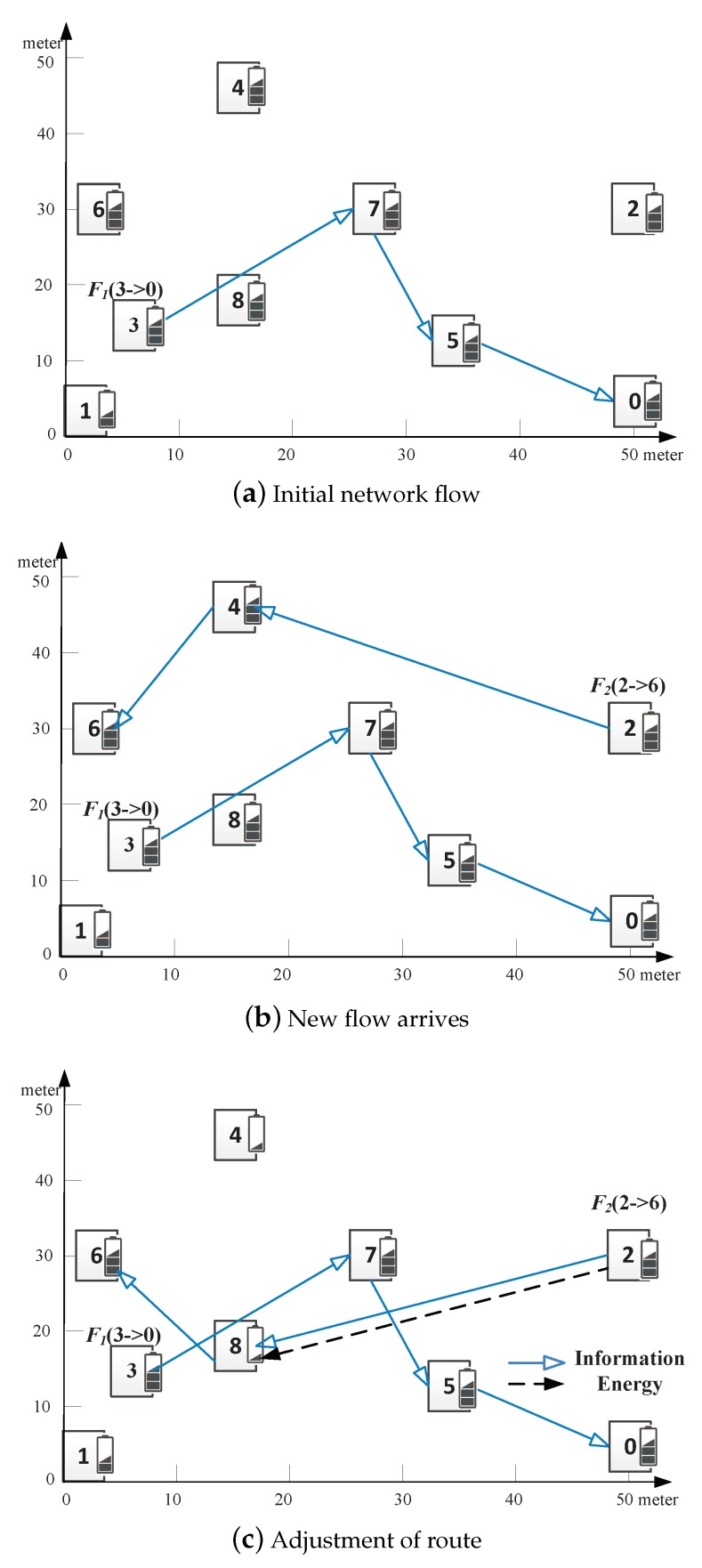
Motivation examples. (**a**) there exists a flow $F_{1}$ from node 3 to node 0 with the path $3\overset{WIT}{\to }7\overset{WIT}{\to }5\overset{WIT}{\to }0$. (**b**) A new flow $F_{2}$ from node 2 to node 6 arrives. Node 4 and path $2\overset{WIT}{\to }4\overset{WIT}{\to }6$ are chosen as the forwarder and the optimal path for flow $F_{2}$, respectively, to avoid interference. (**c**) When the residual energy of nodes 4 and 8 are low than $E_{min}$, the link from node 2 to node 4 cannot build. Therefore, flow $F_{2}$ finds other path $2\overset{SWIPT}{\to }8\overset{WIT}{\to }6$.

A path may be a combination of SWIPT link and WIT link. For example, the first hop $l_{28}^{SWIPT}$ is SWIPT link, while the second hop $l_{86}^{WIT}$ is WIT link for flow $F_{2}\left(2\to 6\right)=2\overset{SWIPT}{\to }8\overset{WIT}{\to }6$, as shown in Figure 5c.

### 3.4. Motivation Example

We take an example to show that interference produces various influences on the performance of multi-flow WSNs with SWIPT.

A multi-flow WSNs with SWIPT consists of 9 sensor nodes as shown in Figure 5. The residual energy is marked on the small bar near the node. In this example, all the SINR, capacity and harvested power of WIT and SWIPT are calculated by Equations (2) and (3) or Equations (5), (7) and (8). We use the same parameters setting in Section 6.

Initially, there exists a flow $F_{1}$ from node 3 to node 0 with the path $3\overset{WIT}{\to }7\overset{WIT}{\to }5\overset{WIT}{\to }0$, as shown in Figure 5a. A new flow $F_{2}$ from node 2 to node 6 arrives.

The interference nodes of node 4 include nodes 2, 3, 6–8. Node 5 does not interfere with node 4 because of barriers. The interference nodes of node 8 include nodes 2–7. Nodes 3, 5, and 7 are on the flow $F_{1}$ which send packets and interfere with flow $F_{2}$. Furthermore, node 8 is closer to those nodes than node 4. The interference power is more serious in node 8 than in node 4. Therefore, node 4 and path $2\overset{WIT}{\to }4\overset{WIT}{\to }6$ are chosen as the forwarder and the optimal path for flow $F_{2}$, respectively, as shown in Figure 5b. According to Equation (3), the capacities of links along the path are $C_{24}^{WIT}=2.65$ Mbps, $C_{46}^{WIT}=2.77$ Mbps. Therefore, the capacity of flow $F_{2}$ is the minimum capacity of links, that is, $\min\{C_{24}^{WIT},C_{46}^{WIT}\}=2.65$ Mbps.

If the residual energy of nodes 4 and 8 are low than $E_{min}$ and they need to be charged when the new flow arrives, the route of flow $F_{2}$ varies. Node 2 does not provide enough energy to node 4 and the link from node 2 to node 4 cannot build. Therefore, the path $2\overset{WIT}{\to }4\overset{WIT}{\to }6$ is not used. Flow $F_{2}$ finds other paths, such as $2\overset{SWIPT}{\to }8\overset{WIT}{\to }6$. The interference power in node 8 is serious which is treated as an energy source. The SWIPT link from node 2 to node 8 build successfully. According to Equation (8), the capacities of links along the path are $C_{28}^{SWIPT}=0.25$ Mbps, $C_{86}^{WIT}=2.77$ Mbps. Therefore, the capacity of flow $F_{2}$ is the minimum capacity of links, that is, $\min\{C_{28}^{SWIPT},C_{86}^{WIT}\}=0.25$ Mbps.

The above example demonstrates that interference plays various roles in the capacity of the link. On one hand, interferences reduce the quality of the link. On the other hand, it provides energy. Therefore, only reducing and avoiding interference is not enough.

### 3.5. Solution Overview

We propose a framework including two important components, namely interference-based information and energy allocation model and interference-aware SWIPT routing to improve the performance.

First, an interference-based information and energy allocation model is designed to analyze the effect of interference and to obtain the maximum capacity of SWIPT link.

Second, to obtain SWIPT and interference gain, the interference and routing metric are calculated by each node. With the help of this metric, when a new flow arrives, we run an interference-aware SWIPT routing algorithm to find the maximum capacity path and allocate information and energy for each hop.

## 4. Interference-Based Information and Energy Allocation

### 4.1. Interference Analysis

**Figure 6:**
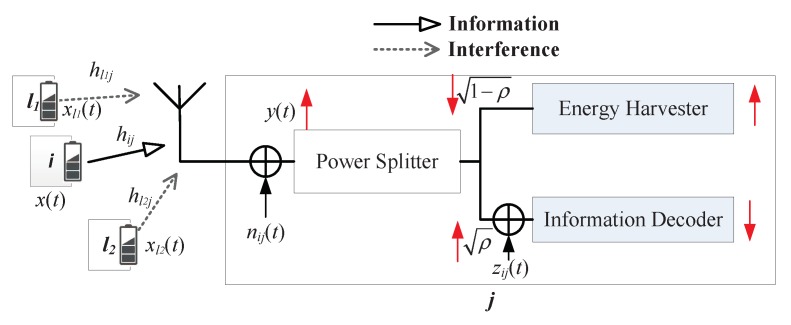
The various influences of interference on SWIPT. The interference reduces the quality of information transmission and becomes a source of energy.

Generally speaking, interference has various influences on SWIPT compared with WIT according to the interference SWIPT mode in Equations (5) and (7). Firstly, interference decreases the SINR in Equation (7) and reduces the quality of information transmission. Secondly, interference improves the power of the received signal, and further increases the received energy in Equation (5), as shown in Figure 6. It means that interference becomes a source of energy. Besides, when the requirement of harvested energy is fixed, the splitting ratio can be lower with more serious interference. Therefore, the SINR varies from splitting ratios to splitting ratios.

In terms of these complex influences, a maximum capacity model is designed to analyze the interference efforts in SWIPT in detail. To improve the performance of a flow, it is useful to maximize the capacity with the interference of links that belong to the flow. For each link, the link capacity depends on the splitting ratio which forms an information and energy allocation problem. Therefore, according to Equations (5), (7) and (8), how to maximize the link capacity with interference can be formulated as follow.

(9) $$\begin{array}{c} \max_{\rho_{ij},P_{ij}}C_{ij}^{\mathrm{SWIPT}}\\ s.t.C_{ij}^{\mathrm{SWIPT}}=W*\log_{2}\left(1+\gamma_{ij}^{\mathrm{ISWIPT}}\right)\\ \;\gamma_{ij}^{\mathrm{ISWIPT}}=\rho_{ij}{\left|h_{ij}\right|}^{2}P_{ij}/\left(\rho_{ij}\sigma_{ij}^{2}+\eta_{ij}^{2}+\rho_{ij}\sum_{l\in \Phi_{j}}P_{l}{\left|h_{lj}\right|}^{2}\right)\\ \;E_{ij}^{Ieh}=\varepsilon \left(1-\rho_{ij}\right)\left(\right|h_{ij}{|}^{2}P_{ij}+\sigma_{ij}^{2}+\sum_{l\in \Phi_{j}}P_{l}{\left|h_{lj}\right|}^{2})\\ \;E_{ij}^{eh}\geq Pc_{j}\\ \;P_{ij}\in \left[0,P_{\max}\right]\\ \;\rho_{ij}\in \left[0,1\right]. \end{array}$$

The model includes three constraints. The harvested energy is higher than the minimum harvesting energy requirement $Pc_{j}$. The value range of transmission power is from 0 to $P_{max}$. The value range of splitting ratio ρ is from 0 to 1. The optimal parameters are the splitting rate and transmission power. The details of how to solve this model are described in Section 4.2.

With the help of this model, the interference effects are analyzed more clearly. According to the setting in Section 6.1, given the sender node, receiver node, and interference node, we vary the interference power from 0 mw to 100 mw. The capacity of SWIPT link is obtained by solving the problem (9). At the same time, we give the capacity of WIT link $C_{ij}^{\mathrm{WIT}}$ with the same interference environment, according to Equations (2) and (3).

**Figure 7:**
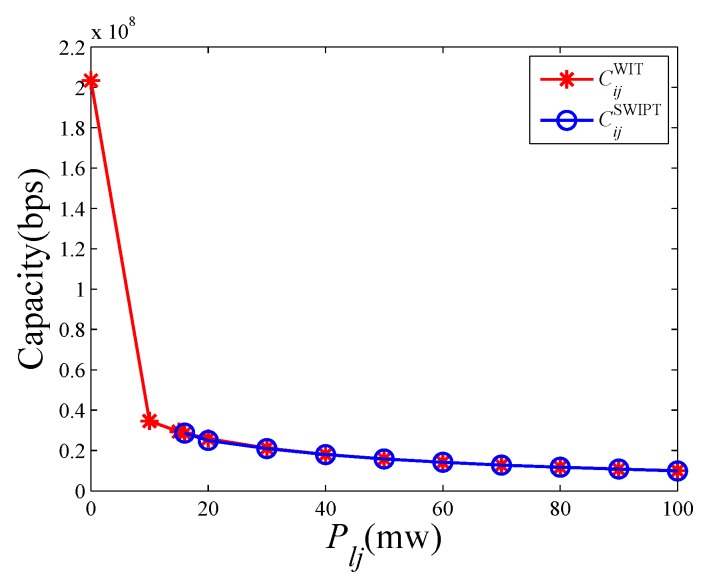
The capacity of WIT and SWIPT under different interference powers.

Figure 7 illustrates that the capacity of WIT and SWIPT vary with different interference powers. As the interference power increases, the link capacities of SWIPT and WIT both become lower. There are two phases. In the first phase, the SWIPT link does not exist when the interference power is lower than 16 mw. In these cases, the received power cannot satisfy the energy need. In the second phase, when the interference power is larger than 16 mw, the received power can satisfy the requirement of energy and the SWIPT link exists. Once the SWIPT link can be set up successfully, the link capacities of SWIPT and IT are equal.

**Figure 8:**
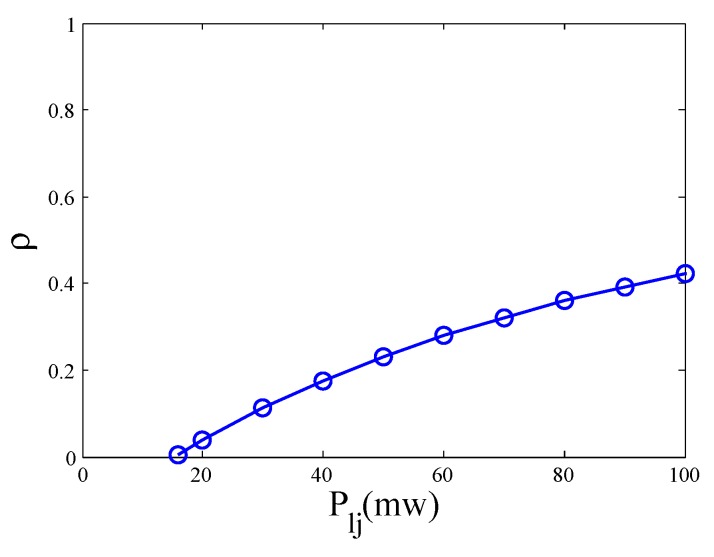
The splitting ratio under different interference powers.

Figure 8 shows the value of power splitting ratio when the maximum capacity is reached with different interference in the second phase. When the SWIPT link is just built, the received signal is only enough to meet the energy harvesting requirement. Most of the signal power is used for energy harvesting, that is, 1−ρ is close to 1 and the power splitting ratio ρ is close to 0. As the interference increases, the received signal power increases. For the same energy harvesting requirement, the ratio for energy harvesting can be reduced and the power splitting ratio increases. Interference is directly proportional to the power splitting ratio.

Noted that, interference can help build a SWIPT link. Once the SWIPT link is built, lower interference is better. Therefore, when the SWIPT link does not exist, selecting the node with higher interference as the next hop to make sure the connectivity. When the SWIPT link is built, selecting the node with lower interference as the next hop to improve the capacity.

### 4.2. Interference-Based Maximum Capacity Information and Energy Allocation Algorithm

We propose a Lagrange multiplier algorithm to solve the problem (9) in this subsection.

The Lagrange function of the problem (9) is obtained in Equation (10) with two Lagrange multipliers a,b.

(10) $$\begin{array}{c} L(\rho_{ij},P_{ij},a,b)\\ =-C_{ij}^{SWIPT}+a\left(Pc_{j}-E_{ij}^{Ieh}\right)+b\left(P_{ij}-P_{\max}\right)\\ =-W*\log_{2}\left(1+\gamma_{ij}^{ISWIPT}\right)+a\left(Pc_{j}-E_{ij}^{eh}\right)+b\left(P_{ij}-P_{\max}\right)\\ =-W*\log_{2}\left(1+\rho_{ij}|h_{ij}{|}^{2}P_{ij}/\left(\rho_{ij}\sigma_{ij}^{2}+\eta_{ij}^{2}+\rho_{ij}\sum_{l\in \Phi_{j}}P_{l}|h_{lj}{|}^{2}\right)\right)+a\left(Pc_{j}-\varepsilon \left(1-\rho_{ij}\right)\left(|h_{ij}{|}^{2}P_{ij}+\sigma_{ij}^{2}+\sum_{l\in \Phi_{j}}P_{l}|h_{lj}{|}^{2}\right)\right)\\ \;+b(P_{ij}-P_{\max}). \end{array}$$

The Lagrange function is convex. This problem can be solved by the Karush-Kuhn-Tucher (KKT) condition. Therefore, we design an iterative interference-based maximum capacity information and energy allocation algorithm in Algorithm 1.

**Algorithm 1** Interference-based maximum capacity information and energy allocation algorithm (capacity).
**Input:** Sender *i*, receiver $j,|h_{ij}{|}^{2},\sigma_{ij}^{2},\eta_{ij}^{2},{Pc}_{j},\sum_{l\in \Phi_{j}}P_{l}{\left|h_{lj}\right|}^{2}$**Output:** 
$\rho_{ij},P_{ij},C_{ij}^{\mathrm{SWIPT}}$

1:Initialization. Set $\rho_{ij}^{0},P_{ij}^{0},a^{1},b^{1}\in R,\mu >0,0\leq \phi \ll 1,\upsilon \in \left(0,1\right),\theta >1,k\leftarrow 1$.2:Based on the $\rho_{ij}^{k-1},P_{ij}^{k-1}$, solve the non-constrained problem (11).
(11)$$\begin{array}{c} \min_{\rho_{ij},P_{ij}}L\left(\rho_{ij},P_{ij},a^{k},b^{k}\right)\\ =-C_{ij}^{SWIPT}+a^{k}\left(Pc_{j}-E_{ij}^{eh}\right)+b^{k}\left(P_{ij}-P_{\max}\right)\\ =-W*\log_{2}\left(1+\gamma_{ij}^{SWIPT}\right)+a^{k}\left(Pc_{j}-E_{ij}^{eh}\right)+b^{k}\left(P_{ij}-P_{\max}\right)\\ =-W*\log_{2}(1+\rho_{ij}|h_{ij}{|}^{2}P_{ij}/\left(\rho_{ij}\sigma_{ij}^{2}+\eta_{ij}^{2}+\rho_{ij}\sum_{l\in \Phi_{j}}P_{l}{\left|h_{lj}\right|}^{2}\right))+a^{k}(Pc_{j}-\varepsilon \left(1-\rho_{ij}\right)\left(\right|h_{ij}{|}^{2}P_{ij}+\sigma_{ij}^{2}+\\ \;\sum_{l\in \Phi_{j}}P_{l}{\left|h_{lj}\right|}^{2}))+b^{k}\left(P_{ij}-P_{\max}\right). \end{array}$$Due to the object function of problem (11) is convex, we find the partial derivative of *L* of $\rho_{ij},P_{ij}$ and let the partial derivative equal zero to get the $\rho_{ij}^{k},P_{ij}^{k}$.
(12)$$\begin{array}{c} P_{ij}^{k}=\left\{\begin{array}{c} \frac{-G_{2}-\sqrt{G_{2}^{2}-4G_{1}G_{3}}}{2G_{1}},G_{1}=0\\ -\frac{G_{3}}{G_{2}},G_{1}\neq 0 \end{array}\right.,\\ \rho_{ij}^{k}=\frac{-G_{7}-\sqrt{G_{7}^{2}-4G_{6}G_{8}}}{2G_{6}},\\ G_{1}=\rho_{ij}^{k-1}|h_{ij}{|}^{2}(a^{k}\left(1-\rho_{ij}^{k-1}\right)|h_{ij}{|}^{2}-b^{k})/\left(\rho_{ij}^{k-1}G_{5}+\eta_{ij}^{2}\right),\\ G_{2}=(a^{k}\left(1-\rho_{ij}^{k-1}\right)|h_{ij}{|}^{2}-b^{k}),\\ G_{3}=W/\ln\left(2\right),\\ G_{4}=a^{k}\varepsilon \left(1-\rho_{ij}\right)\left(\right|h_{ij}{|}^{2}P_{ij}^{k-1}+\sigma_{ij}^{2}+\sum_{l\in \Phi_{j}}P_{l}{\left|h_{lj}\right|}^{2}),\\ G_{5}=\sigma_{ij}^{2}+\sum_{l\in \Phi_{j}}P_{l}{\left|h_{lj}\right|}^{2},\\ G_{6}=G_{5}\left(G_{5}+\sum_{l\in \Phi_{j}}P_{l}{\left|h_{lj}\right|}^{2}\right),\\ G_{7}=\eta_{ij}^{2}\left(G_{5}+\sum_{l\in \Phi_{j}}P_{l}{\left|h_{lj}\right|}^{2}\right)+G_{5}-G_{3}G_{5}\sum_{l\in \Phi_{j}}P_{l}{\left|h_{lj}\right|}^{2}/G_{4},\\ G_{8}=\eta_{ij}^{2}\left(1-G_{3}\sum_{l\in \Phi_{j}}P_{l}{\left|h_{lj}\right|}^{2}/G_{4}\right) \end{array}$$3:Stop criterion. If $|L^{k}-L^{k-1}|\leq \phi$, return $\rho_{ij}^{k},P_{ij}^{k}$.4:Update μ, if $|L^{k}\left|\right|\geq \upsilon ||L^{k-1}\left|\right|$, μ:=θμ.5:Update lagrange multipliers.
(13)$$\begin{array}{c} a^{k+1}=\max\{0,a^{k}+\mu (Pc_{j}-\varepsilon \left(1-\rho_{ij}^{k}\right)\left(\right|h_{ij}{|}^{2}P_{ij}^{k}+\sigma_{ij}^{2}+\sum_{l\in \Phi_{j}}P_{l}{\left|h_{lj}\right|}^{2}))\}\\ b^{k+1}=\max\left\{0,b^{k}+\mu \left(P_{ij}^{k}-P_{\max}\right)\right\} \end{array}$$6:k←k+1, goto step 2.


Initially, all parameters are set at random. In every iteration, sensor node *j* solves the non-constrained problem (11) by KKT condition to obtain the result in Equation (12). The loop stops when the stop criterion is satisfied. Otherwise, we update the parameter μ and the Lagrange multipliers $a^{k+1},b^{k+1}$ by (13) based on the parameters in the previous iteration.

## 5. Interference-Aware SWIPT Routing

In this section, an interference-aware routing metric and an interference-aware SWIPT routing algorithm are proposed to evaluate the capacity of links and path, and take full use of SWIPT with interference.

### 5.1. Interference-Aware Routing Metric

According to the above interference-based allocation model, we design an interference-aware routing metric and formulate the routing problem. In multi-hop WSNs, two kinds of transmission, such as WIT and SWIPT, are both available. The link can work at any one transmission mode. The transmission mode with higher capacity should be better. Therefore, we define the routing metric of link (inference-aware capacity available metric (IaCA)) by the maximum link capacity between WIT and SWIPT based on Equations (3) and (8).

(14) $${\mathrm{IaCA}}_{ij}=\max\left\{C_{ij}^{WIT},C_{ij}^{SWIPT}\right\}.$$

The path metric is the minimum capacity among the link metrics of all links along the path.

(15) $${\mathrm{IaCA}}_{sd}=\min\left\{{\mathrm{IaCA}}_{sc},{\mathrm{IaCA}}_{cd}\right\},c\in {Path}_{sd}$$

where *c* is the node in the path from *s* to *d*. According to the allocation model and flow conservation, the problem of selecting a path from $s_{k}$ to $d_{k}$ can be formulated as the problem (16).

(16) $$\begin{array}{c} \max_{r,P,\rho }\min r_{ij}{\mathrm{IaCA}}_{\mathrm{ij}}\\ s.t.{C1:\mathrm{IaCA}}_{\mathrm{ij}}=\max\left\{C_{ij}^{WIT},C_{ij}^{SWIPT}\right\},\forall i,j\\ C2:C_{ij}^{WIT}=W*\log_{2}\left(1+\gamma_{ij}^{IWIT}\right),\forall i,j\\ C3:\gamma_{ij}^{IWIT}={\left|h_{ij}\right|}^{2}P_{ij}/\left(\sigma_{ij}^{2}+\eta_{ij}^{2}+\sum_{l\in \Phi_{j}}P_{l}{\left|h_{lj}\right|}^{2}\right),\forall i,j\\ C4:C_{ij}^{SWIPT}=W*\log_{2}\left(1+\gamma_{ij}^{ISWIPT}\right),\forall i,j\\ C5:\gamma_{ij}^{ISWIPT}=\rho_{ij}{\left|h_{ij}\right|}^{2}P_{ij}/\left(\rho_{ij}\sigma_{ij}^{2}+\eta_{ij}^{2}+\rho_{ij}\sum_{l\in \Phi_{j}}P_{l}{\left|h_{lj}\right|}^{2}\right),\forall i,j\\ C6:E_{ij}^{Ieh}=\varepsilon \left(1-\rho_{ij}\right)\left(\right|h_{ij}{|}^{2}P_{ij}+\sigma_{ij}^{2}+\sum_{l\in \Phi_{j}}P_{l}{\left|h_{lj}\right|}^{2}),\forall i,j\\ C7:E_{ij}^{Ieh}\geq Pc_{j},\mathrm{if}E_{j}<E_{\min},\forall i,j\\ C8:Pc_{j}=P_{jk},\mathrm{if}r_{jk}=1,\forall j\\ C9:\sum_{j}r_{ij}-\sum_{j}r_{ji}=\left\{\begin{array}{c} 1,i=s.\\ -1,i=d.\\ 0,other. \end{array}\right.,\forall i\\ C10:0\leq P_{ij}\leq P_{\max},\forall i,j\\ C11:\rho_{ij}\in \left[0,1\right],\forall i,j\\ C12:r_{ij}\in \left\{0,1\right\},\forall i,j\\ C13:i,j\in [1\ldots N], \end{array}$$

where $r_{ij}$ is a binary variable with 1 when the link $l_{ij}$ is passed by the data of flow, and with 0 when not. C1 constrain is the routing metric Equation (15). C2,C3 constrains are the capacity of link with WIT Equations (2) and (3), and C4,C5,C6 constrains are the capacity of link with SWIPT Equations (5), (7) and (8). The C7 constrain is the energy harvesting requirement when nodes has not enough energy. C8 constrain shows that the energy harvesting requirement for node *j* is determined by the transmission power to next-hop node *k*. C9 constrain is the flow conservation to find a path from *s* to *d*. C10−C13 constrains are the range of parameters. We can solve problem (16) to get the route from $s_{k}$ to $d_{k}$.

When the node’s residual energy is enough or higher than the minimum energy requirement $E_{min}$, it is not necessary to replenishing energy. Therefore, the minimum harvesting energy requirement $Pc_{j}$ is zero and the power splitting ratio ρ can be set to 1. The maximum link capacity of SWIPT and WIT are equal. When the node’s residual energy is lower than $E_{min}$, the node needs to replenish energy. The problem (16) can be transformed to problem (17).

(17) $$\begin{array}{c} \max_{P,r,\rho }\min r_{ij}C_{ij}^{SWIPT}\\ s.t.C4:C_{ij}^{SWIPT}=W*\log_{2}\left(1+\gamma_{ij}^{ISWIPT}\right),\forall i,j\\ C5:\gamma_{ij}^{ISWIPT}=\rho_{ij}{\left|h_{ij}\right|}^{2}P_{ij}/\left(\rho_{ij}\sigma_{ij}^{2}+\eta_{ij}^{2}+\rho_{ij}\sum_{l\in \Phi_{j}}P_{l}{\left|h_{lj}\right|}^{2}\right),\forall i,j\\ C6:E_{ij}^{Ieh}=\varepsilon \left(1-\rho_{ij}\right)\left(\right|h_{ij}{|}^{2}P_{ij}+\sigma_{ij}^{2}+\sum_{l\in \Phi_{j}}P_{l}{\left|h_{lj}\right|}^{2}),\forall i,j\\ C14:E_{ij}^{Ieh}\geq \left\{\begin{array}{c} 0,E_{j}\geq E_{\min}\\ Pc_{j},E_{j}<E_{\min} \end{array}\right.,\forall i,j\\ C8:Pc_{j}=P_{jk},\mathrm{if}r_{jk}=1,\forall j\\ C9:\sum_{j}r_{ij}-\sum_{j}r_{ji}=\left\{\begin{array}{c} 1,i=s.\\ -1,i=d.\\ 0,other. \end{array}\right.,\forall i\\ C10:0\leq P_{ij}\leq P_{\max},\forall i,j\\ C11:\rho_{ij}\in \left[0,1\right],\forall i,j\\ C12:r_{ij}\in \left\{0,1\right\},\forall i,j\\ C13:i,j\in [1\ldots N], \end{array}$$

where the optimal variables are P,r,ρ. Virtual link method can not be exploited by this problem (17) directly due to the minimum harvesting energy requirement $Pc_{j}$ determined by the node’s residual energy and next-hop selection. An interference-aware SWIPT routing algorithm is proposed for this problem in the following subsection.

### 5.2. Interference-Aware SWIPT Routing Algorithm

The main idea of our interference-aware SWIPT routing algorithm (ISWIPTR) is to select a node’s next-hop before allocating information and energy for the node as receiver. In this way, allocating information and energy can be independent from the ISWIPTR. The pseudo code of ISWIPTR is shown in Algorithm 2 which searches the maximum capacity path by allocating the information and energy to select link with Algorithm 1.

We modify dijkstra routing [11,23,51] to design ISWIPTR. The inputs are graph G(V,E), source $s_{k}$ and destination $d_{k}$ of flow $F_{k}$, the information of the previous flows which includes the path of the flows $F_{1},\cdots ,F_{k-1}$. The outputs are the selected flow $F_{k}$’s path with capacity and $\rho_{ij},P_{ij}$ of each hop (i,j). ${\mathrm{IaCA}}_{i}^{k}$ represents metric of the maximum capacity path from *i* to destination $d_{k}$. The next-hop node of node *i* to destination $d_{k}$ is stored on $F_{i}^{k}$. $Pc_{i}$ is the energy harvesting power requirement of node *i* for forwarding data to next-hop $F_{i}^{k}$. Nodes which already have a maximum capacity path are included by a set *S*. All nodes without a maximum capacity path is included in *Q*. *Q* is a priority queue and the key is ${\mathrm{IaCA}}_{i}^{k}$.

The route metric value, energy harvesting power requirement, and next-hop of nodes are initial in lines 1–5. The route metric value of destination is initial to zero, *S* is empty, and queue *Q* includes all the nodes in lines 6–9. In the while loop, the node with the minimum IaCA in *Q* is selected, denoted by *j*. Once node *j* is selected, the maximum path from node *j* to destination $d_{k}$ has found. For every link (i,j)∈E, firstly, the interference from the previous flows is obtained by the Algorithm 3. Secondly, the maximum capacity with SWIPT is calculated by the Algorithm 1. Thirdly, the number of links that share nodes with link (i,j) is got by the Algorithm 4. No matter that the link (i,j) is SWIPT mode or WIT mode, the links sharing nodes each other should share channel according to the TDMA. Therefore, the available capacity equals the maximum capacity divided by the number of link-sharing nodes with link (i,j). The node *i*’s temporary route metric ${\mathrm{IaCA}}_{i}^{k^{\prime }}$ can be calculated by the minimum of ${\mathrm{IaCA}}_{ij}^{k}$ and ${\mathrm{IaCA}}_{j}^{k}$. In lines 19–25, when temporary route metric is higher than the route metric of node *i*, node *j* is selected as the next-hop $F_{i}^{k}$ and the route metric ${\mathrm{IaCA}}_{i}^{k}$ is updated by the temporary route metric. When node *i*’s residual energy is lower than $Er_{min}$, the harvesting power requirement $Pc_{i}$ is updated by $P_{ij}$. In the next iterations of while loop, the forwarding power requirement $Pc_{i}$ may be needed in Algorithm 1 inline 15 which is obtained when settling node *i*. All nodes find the path to destination $d_{k}$ till the queue *Q* is empty.

**Algorithm 2** Interference-aware SWIPT routing algorithm.
**Input:** 
$G\left(V,E\right),\left(s_{k},d_{k}\right),flows$
**Output:** flow $F_{k}$’s path with capacity and $\rho_{ij},P_{ij}$ of each hop (i,j)
1:**for** every node *i* in V **do**2:  ${\mathrm{IaCA}}_{i}^{k}\leftarrow \infty$3:  $Pc_{i}^{k}$← 04:  $F_{i}^{k}$← NIL5:
**end for**
6:
${\mathrm{IaCA}}_{d_{k}}^{k}\leftarrow 0$
7:
$Pc_{d}^{k}\leftarrow 0$
8:
S←∅
9:
Q←V
10:**while**Q≠ ∅ **do**11:  *j*← EXTRACT-MIN(*Q*)12:  S←S⋃ {*j*}13:  **for** every link (i,j)∈E **do**14:    $\sum_{l\in \Phi_{j}}P_{l}{\left|h_{lj}\right|}^{2}\leftarrow InterferenceSum\left(i,j,flows\right)$ with Algorithm 315:    $\rho_{ij}^{k},P_{ij}^{k},C_{ij}^{SWIPT,k}\leftarrow Capacity\left(i,j,|h_{ij}{|}^{2},\sigma_{ij}^{2},\eta_{ij}^{2},Pc_{j}^{k},\sum_{l\in \Phi_{j}}P_{l}{\left|h_{lj}\right|}^{2}\right)$ with Algorithm 116:    cointer←Cointerference(i,j,flows,k) with Algorithm 417:    ${\mathrm{IaCA}}_{ij}^{k}\leftarrow C_{ij}^{SWITP,k}/\left(1+cointer\right)$18:    ${\mathrm{IaCA}}_{i}^{k^{\prime }}\leftarrow \min\left({\mathrm{IaCA}}_{ij}^{k},{\mathrm{IaCA}}_{j}^{k}\right)$19:    **if** ${\mathrm{IaCA}}_{i}^{k^{\prime }}>{\mathrm{IaCA}}_{i}^{k}$ **then**20:      ${\mathrm{IaCA}}_{i}^{k}\leftarrow {\mathrm{IaCA}}_{i}^{k^{\prime }}$21:      $F_{i}^{k}\leftarrow j$22:      $\rho_{i}^{k}\leftarrow \rho_{ij}^{k}$23:      $P_{i}^{k}\leftarrow P_{ij}^{k}$24:      $Pc_{i}^{k}\leftarrow E_{i}<E_{min}?P_{ij}^{k}:0$25:    **end if**26:  **end for**27:
**end while**



Link (i,j)’s interference caused by the previous flows is calculated in Algorithm 3. A node *l* interferes with a link (i,j) only if node *l* is within the two-hop range of node *j*. The interference range is about twice that of transmission range under the IEEE 802.11 WLAN standard. Once a flow passes node *l* and the next-hop of node *l* is not node *i* and *j*, the signal send by node *l* should interfere with link (i,j). For simplicity, the maximum transmission power is selected as the interference power when multiple flows pass node *l*. Additionally, the interference generated by all the nodes is accumulated, that is, the product of the interference power and the channel gain from *l* to *j* is summed.

The interference caused by the node *i* and *j* is not considered in Algorithm 3. A previous flow passes the node *i* or *j*, there is no interference between the two flows but they cannot work simultaneously. According to the TDMA, the links should share the channel with each other when they share nodes with each other. Therefore, the available capacity equals the maximum capacity divided by the number of links sharing nodes with link (i,j).

**Algorithm 3** Interference Algorithm (InterferenceSum).
**Input:** 
i,j,flows
**Output:** 
$\sum_{l\in \Phi_{j}}P_{l}{\left|h_{lj}\right|}^{2}$

1:**for** every node *l* in V **do**2:  **if** l≠i&&l≠j&&l is the two hop neighbor of *j* **then**3:    **for** each flow *f* in flows **do**4:      **if** $P_{l}^{f}>P_{l}\&\&F_{l}^{k}\neq i\&\&F_{l}^{k}\neq j$ **then**5:        $P_{l}\leftarrow P_{l}^{f}$6:      **end if**7:    **end for**8:    $interference+=P_{l}{\left|h_{lj}\right|}^{2}$9:  **end if**10:
**end for**
11:return interference


**Algorithm 4** Interference link number Algorithm (Cointerference).
**Input:** 
i,j,flows,k
**Output:** 
cointerference_number

1:
cointerference_number=0
2:**for** each flow *f* in flows **do**3:  **if** i∈f **then**4:    $cointerference\_number+=(i==s_{f}||i==d_{f}?1:2)$5:  **end if**6:  **if** j∈f **then**7:    $cointerference\_number+=(j==s_{f}||j==d_{f}?1:2)$8:  **end if**9:  **if** i∈f&&j∈f **then**10:    cointerference_number−−11:  **end if**12:
**end for**
13:
**if** $i==s_{k}\left|\right|j==d_{k}$ **then**
14:  cointerference_number+=115:
**else if** $i==s_{k}\&\&j==d_{k}$ **then**
16:  cointerference_number+=017:
**else**
18:  cointerference_number+=219:
**end if**
20:return cointerference_number


There are three cases according to the relationship of nodes.

- In the first case, the previous flow only passes the sending node. As shown in Figure 5a, flow $F_{1}$ pass node 7 for link (7,2). Two links (3,7) and (7,5) share the node 7 with link (7,2). For link (3,4), flow $F_{1}$ pass node 3 and node 3 is the source node of flow $F_{1}$. Therefore, only one link (3,7) share the node 3 with link (3,4).
- In the second case, the previous flow only passes the receiving node. As shown in Figure 5b, the number of shared links is 1 if the receiver node is the source node or destination node of the previous flow; the number is 2 if not.
- In the last case, the previous flow passes both the sender node and receiver node. As shown in Figure 5c, the shared links are the union of the two former cases. The link (i,j) is involved in the first and second cases. Therefore, the number of shared links is the sum of the first and second cases minus one.

The interference caused by the current flow of $F_{k}$ also needs to be taken into account. Similarly, there are three cases.

- The sending node is the source node or the receiver node is the destination node of flow $F_{k}$. The link (i,j) has no previous link or next link. The number of shared links in flow $F_{k}$ is 1.
- The sending node is the source node and the receiver node is the destination node of flow $F_{k}$. The link (i,j) has no previous link and next link. No link shares a node with it in flow $F_{k}$.
- Neither the sending node is the source node nor the receiver node is the destination node of flow $F_{k}$. The link (i,j) has a previous link and a next link. The number of shared links in flow $F_{k}$ is 2.

### 5.3. Computation Complexity Analysis

The Algorithm 2 uses the Algorithms 1, 3 and 4. Therefore, we analyze the Algorithms 1, 3 and 4 first. In Algorithm 1, the problem 10 is solved directly where the time complexity is O(1). The time complexity depends on the convergence. We assume that after *I* iterations, the algorithm will stop. The time complexity is O(I). In Algorithm 3, each node is checked whether it is the interference node of the receiver node *j*. When yes, the transmission power of each existed flow on sender *i* are compared to select the maximum power. Therefore, the time complexity is O(|V|(k−1)), where |V| is the number of nodes and *k* is id of current flow to find path. In Algorithm 4, each existed flow check the relationship between the flow path and link. Therefore, the time complexity is O(k−1). In Algorithm 2, the time complexity of the initial phase is O(|V|). In |V| iterations, the node *j* with the smallest weight is selected, which costs O(|V|). For each link of node *j*, the Algorithms 1, 3 and 4 are called. Therefore, the total time complexity is $O(|V|+\left|V|(|V\right|+D(|V|\left(k-1\right)+I+\left(k-1\right))))=O(|V|+{\left|V\right|}^{2}+{\left|V\right|}^{2}Dk+|V|DI+\left|V|Dk\right)$, where *D* is the maximum degree of all nodes.

## 6. Simulation Results and Analysis

We compare and evaluate the performance of our scheme by comparing with three other different schemes. The first scheme uses WIT and the Equation (3) without the interference part as the route metric, which is named by WIT without interference (WITwoi). The second scheme uses the maximum capacity between WIT and SWIPT as route metric, but there are no interference parts in the Equation (3) and Equation (8), which is named by SWIPT without interference (SWIPTwoi). The third scheme uses Equation (3) as the route metric which is named by WIT with interference (WITwi). The last one is our scheme denoted by SWIPTwi.

We take the Figure 5 as the simulation network topology which includes 9 nodes. we assume that the system satisfies the full energy of node $Er_{full}=1$, the minimum energy requirement for forwarding $Er_{min}=0.4$, the maximum transmission power $P_{max}=100$ mw, the minimum harvesting energy requirement $Pc_{j}=10$ mW, and the energy converting coefficient ε=0.65.

The remaining energy of 1, 3, 4, and 8 nodes is insufficient to forward data which are lower than $Er_{min}$. They need to replenish energy. According to the setting [16,17], the channel model is described as $|h_{ij}{|}^{2}={1/(1+||i-j||}^{\alpha })$, where ||i−j|| represents the distance between node *i* and node *j*, and the path-loss exponent α is set to 2.7 for urban cellular communication environment. The channel bandwidth *W* is set to $10^{6}$. It is assumed that all nodes are in the same noise environment [34], i.e., $\sigma_{ij}^{2}=\sigma^{2},\eta_{ij}^{2}=\eta^{2}$. The power of noises are set to $\sigma^{2}=-50$ dBm, $\eta^{2}=-70$ dBm. Table 2 lists all the simulation parameters setting.

First, we analyze the sensitiveness of energy converting coefficient ε. Next, we characterize the effect of flows. Because there is no interference with single flow, the number of flows is set from two to four. We select 50 source and destination nodes pairs for each number of flows at random. Two metrics, such as gain distribution and capacity, are taken into account to evaluate the performance. Gain distribution is the percentage of flows which can take SWIPT or interference to improve performance. Capacity is the path capacity of the last flow, that is, the route metric of the last flow.

### 6.1. The Impact of Energy Converting Coefficient

Many references [6,39,52] assume that ε is 1 for the convenience of analysis, but it can not be realized in practice. According to Intel, wireless recharging is effective for transferring 60 watts of power over a distance of up to two to three meters with an efficiency of 75 percent [53]. We vary the ε from 0.5 to 1 and illustrate the capacity of the SWIPT and splitting ratio. There are a sender, a receiver and an interference node. The distance between the sender and receiver, between the interference node and receiver are 2 m. The minimum harvesting energy requirement of the receiver was 10 mW. The transmitting power of the interference node increases from 0 mw to maximum power 100 mw.

**Figure 9:**
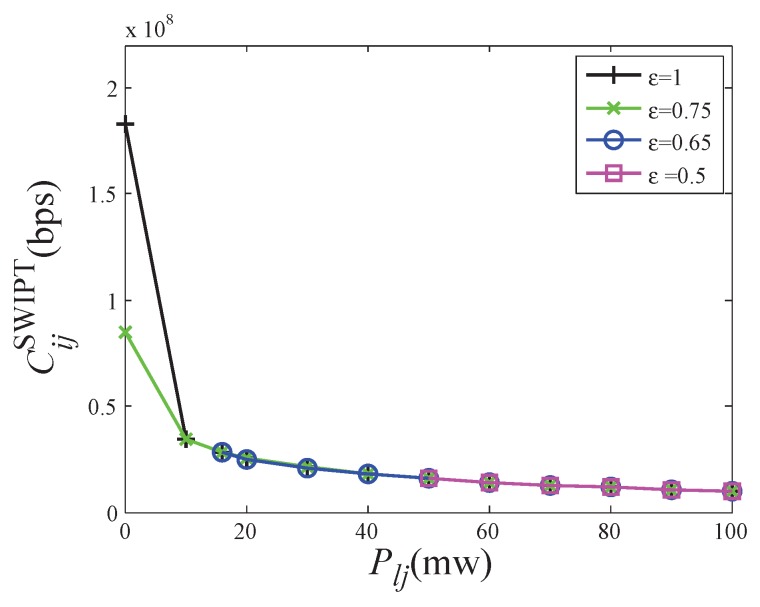
The impact of energy converting coefficient on SWIPT capacity.

Figure 9 illustrates the capacity of SWIPT varies with different energy converting coefficients and interference powers. As the energy converting coefficient decreases, the SWIPT link needs higher interference power to be set up. For example, the SWIPT link exists without interference when the energy converting coefficient is 1 or 0.75. The SWIPT link does not exist until the interference power increases to 50mv when the energy converting coefficient is 0.5. Once the SWIPT link can be set up successfully, the SWIPT link capacities with all energy converting coefficients are equal.

**Figure 10:**
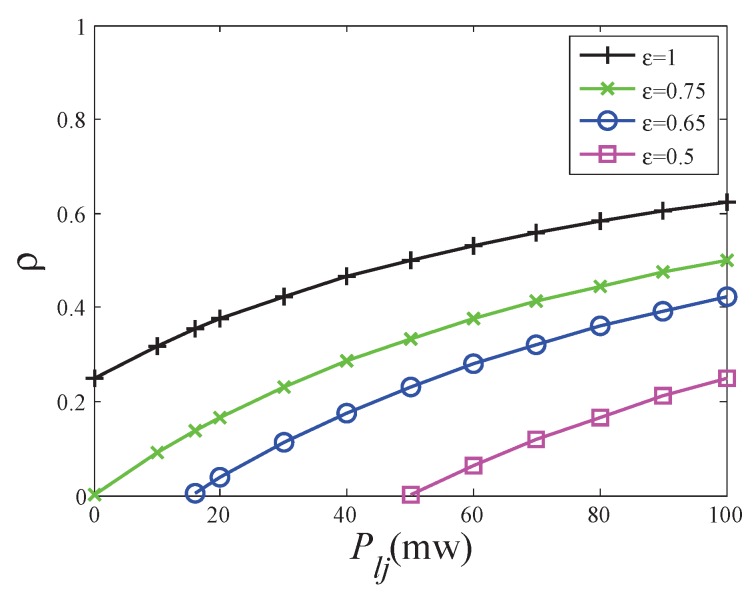
The impact of energy converting coefficient on splitting ratio.

Figure 10 shows the value of the power splitting ratio when the maximum capacity is reached with different energy converting coefficients and interference powers. As the energy converting coefficient decreases, the maximum capacity is reached with lower the power splitting ratio at the same interference power. It means that more received power is used for energy harvesting because of the lower energy converting coefficient. Promoting the energy converting coefficient can take full use of power resources.

### 6.2. Gain Distribution

According to the related work [17], we know that according to the distribution of source and destination of flow, not all flows can take SWIPT to improve performance. Therefore, we analyze the gain distributions of the 50 different flows. Regardless of the number of flows, the flows can be divided into three categories.

- In the first category, the path capacities of the four schemes are equal, because the path selected by the proposed scheme is the same as the other three schemes.
- In the second category, the path capacities of the schemes which take account of interference, such as WITwi and SWIPTwi, are higher than that of the schemes without interference, such as WITwoi and SWIPTwoi. However, the performance of WITwi is equal to that of SWIPTwi. The paths selected by WITwi and SWIPTwi are the same, which are different from the paths of WITwoi and SWIPTwoi. In this case, SWIPT does not improve the performance.
- In the third category, the performance of SWIPTwi is higher than that of WITwoi, SWIPTwoi, and WITwi. The routing result of SWIPT with interference differs from that of WIT with interference. In this case, it can be seen that the performance is improved by SWIPT.

In other words, the path selected by the proposed scheme may be the same as the other three schemes. The first category and the second category are these kinds of situations. Therefore, these three categories are named by no gain, gain from interference, and gain from SWIPT.

**Figure 11:**
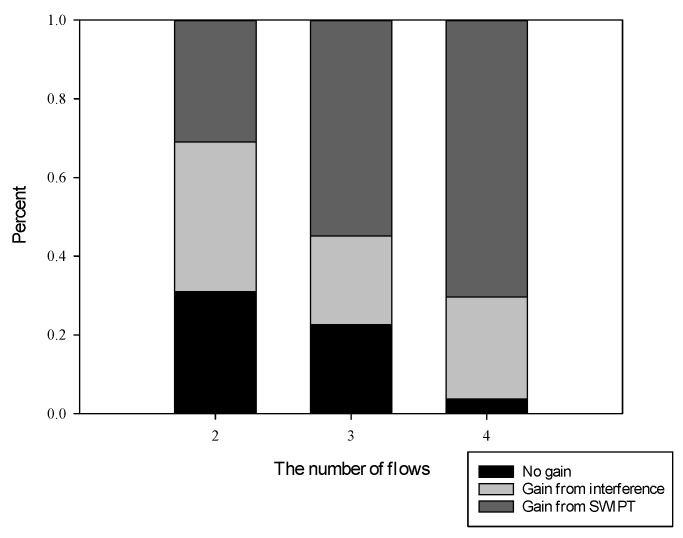
Categories distribution with the different number of flows.

Figure 11 shows the proportion of three categories with the number of flows. It can be seen that the sum of the third category and the second category is greater than 65% in each number of flows. The proportion of the first category decreases significantly as the number of flows increases. The proportion of second and third category grows up to above 95% when the number of flows is 4. The proportion of the third category increases from 30% to 70%. It means that as the number of flows increases, there is more likely to obtain performance gains from interference and SWIPT.

### 6.3. Impact of Flow

According to the gain distribution, above 65% flows can obtain gain from interference and SWIPT. How the interfere and SWIPT impact the network performance are analyzed in detail. We analyze the average capacity of four schemes. The average capacity is the average capacity of flows that belong to the same category.

**Figure 12:**
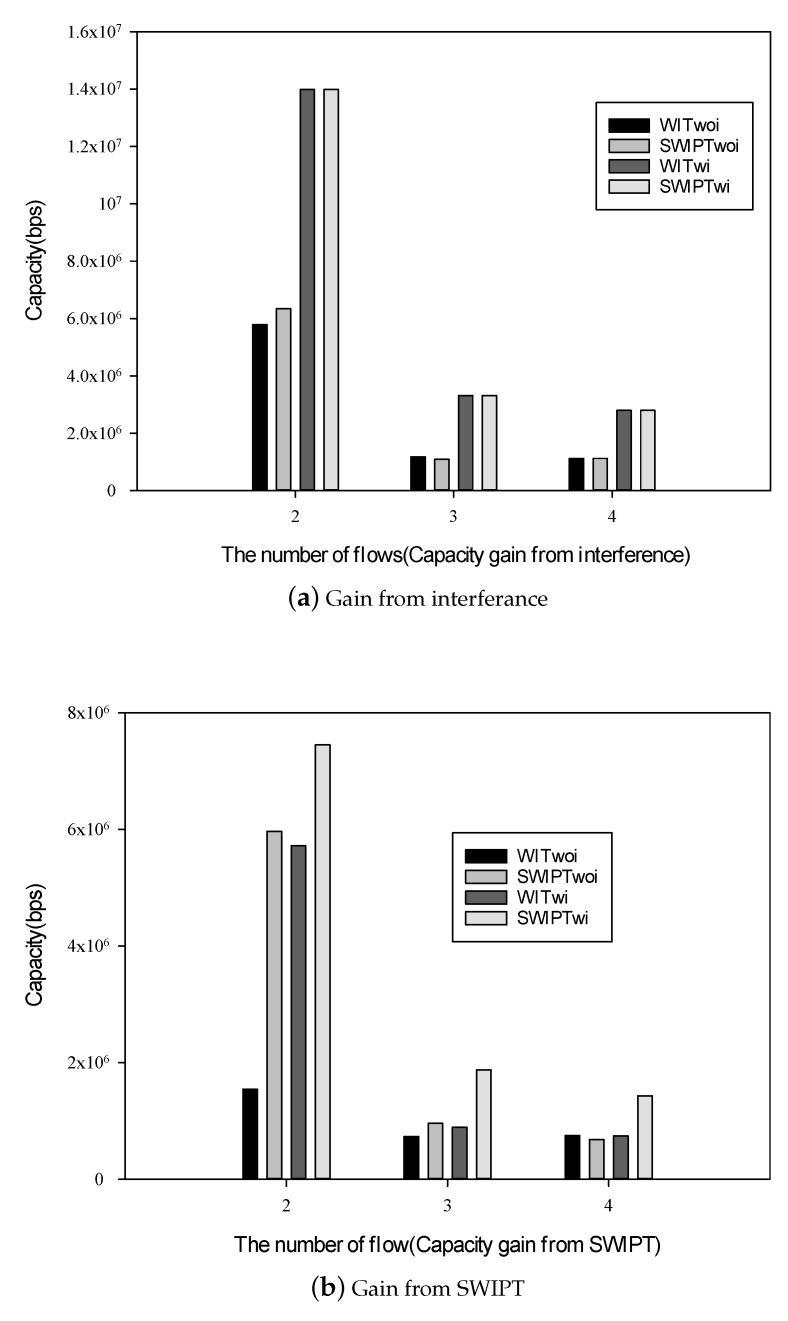
Capacity with different categories.

The average capacity of the second category is shown in Figure 12a. The average capacity of schemes considering interference, such as WITwi and SWIPTwi, are 1.4 times as high as that of schemes without interference, such as WITwoi and SWIPTwoi. This benefit is from taking account of interference.

The total capacity resource is limited and needs to be shared by all flows. Therefore, the average values of path capacity in the low regime of the number of flow must be higher than that in the high regime of the number of flows. When the number of flows is in the low regime, the capacity can not be fully utilized by a few flows and suitable interference and SWIPT design can improve the path capacity significantly. When the number of flows is in the high regime, the capacity is fully shared by several flows and the rest capacity exploited by suitable interference and SWIPT design is not too much. Therefore, the differences among the four schemes in the low regime of the number of flow are wide than that in the high regime of the number of flow.

The average capacity of the third category is shown in Figure 12b. The third category is benefits from using SWIPT. The average capacity of SWIPTwi is 90–382% higher than that of SWIPTwoi and 30–110% higher than that of WITwi, as shown in Figure 12b.

## 7. Conclusions

To solve the multi-flow routing in wireless sensor network with SWIPT, we analyze the influence of interference on SWIPT. By jointly considering interference, SWIPT, and routing, we design an interference-based information and energy allocation model to maximize the link capacity with SWIPT, formulate an interference-aware SWIPT routing problem and design algorithm for the routing problem. Our solution effectively takes full use of the SWIPT technique and interference to improve the capacity of new flow with maximum capacity information and energy allocation and routing selection. Our research results reflect a well-designed scheme can obtain capacity performance gains from interference and SWIPT.

## Figures and Tables

**Table 1:** Summary of notations.

Notation	Definition
$Er_{i}$	Residual energy of node *i*
$Er_{min}$	Minimum energy requirement for forwarding
$P_{ij}$	Transmission power from node *i* to node *j*
x(t)	send signal
y(t)	received signal
$y^{EH}\left(t\right)$	received signal used for the energy harvesting circuit
$y^{ID}\left(t\right)$	received signal used for the information decoding circuit
$P_{l}$	interference power from node *l*
$\Phi_{j}$	node set interfered with node *j*
$|h_{ij}{|}^{2}$	Propagation power gain from node *i* to node *j*
$\sigma_{ij}^{2}$	Power of antenna noise from node *i* to node *j*
$\eta_{ij}^{2}$	Power of signal conversion noise from node *i* to node *j*
$\gamma_{ij}^{IWIT}$	Signal-to-noise rate for WIT
$\gamma_{ij}^{ISWIPT}$	Signal-to-noise rate for SWIPT
$C_{ij}^{WIT}$	Capacity for WIT
$C_{ij}^{SWIPT}$	Capcity for SWIPT
$\rho_{ij}$	Power fraction for decoding information from node *i* to node *j*
$1-\rho_{ij}$	Power fraction for harvesting energy from node *i* to node *j*
$E_{ij}^{eh}$	Energy harvesting power from node *i* to node *j*
ε	Energy converting coefficient
$Pc_{j}$	Minimum harvesting power requirement of node *j*
$P_{max}$	Maximum transmission power
α	Path-loss exponent
*W*	The fading channel bandwidth

**Table 2:** Simulation parameters.

Parameters	Values
$Er_{full}$	1
$Er_{min}$	0.4
$P_{max}$	100 mW
$Pc_{j}$	10 mW
$|h_{ij}{|}^{2}$	$1/(1+||i-j|{|}^{\alpha })$
α	2.7
$\sigma_{ij}^{2}$	−50 dBm
$\eta_{ij}^{2}$	−70 dBm
ε	0.65
*W*	$10^{6}$

## Abbreviations

The following abbreviations are used in this manuscript:

SWIPT	Simultaneous wireless information and power transfer
WIT	Wireless information transmission
RF	Radio frequency
TS	Time switching
PS	Power splitting
CSCG	Circularly symmetric complex Gaussian
ID	Information decoding
SINR	Signal-to-interference-plus-noise ratio
EH	Energy harvesting
ISWIPTR	Inference-aware capacity available metric
WITwoi	WIT without interference
SWIPTwoi	SWIPT without interference
WITwi	WIT with interference
SWIPTwi	SWIPT with interference

## References

[1] Varshney L.R. Transporting Information and Energy Simultaneously. Proceedings of the IEEE International Symposium on Information Theory (ISIT).

[2] Li W., Chen Z., Gao X., Liu W., Wang J. (2019). Multi-Model Framework for Indoor Localization under Mobile Edge Computing Environment. IEEE Internet Things J..

[3] Yin Y., Xu Y., Xu W., Min G., Pei Y. (2017). Collaborative Service Selection via Ensemble Learning in Mixed Mobile Network Environments. Entropy.

[4] Nguyen T., Pan J., Dao T. (2019). An Improved Flower Pollination Algorithm for Optimizing Layouts of Nodes in Wireless Sensor Network. IEEE Access.

[5] Valenta C., Durgin G. (2014). Harvesting wireless power: Survey of energy-harvester conversion efficiency in far-field, wireless power transfer systems. IEEE Microw. Mag..

[6] Zhang R., Ho C.K. (2013). MIMO Broadcasting for Simultaneous Wireless Information and Power Transfer. IEEE Trans. Wirel. Commun..

[7] Liu L., Zhang R., Chua K.C. (2013). Wireless Information and Power Transfer: a Dynamic Power Splitting Approach. IEEE Trans. Commun..

[8] Zhou X., Zhang R., Ho C.K. (2013). Wireless Information and Power Transfer: Architecture Design and Rate-energy Tradeoff. IEEE Trans. Commun..

[9] Luo Y., Yang K., Tang Q., Zhang J., Li P., Qiu S. (2016). An optimal data service providing framework in cloud radio access network. Eurasip J. Wirel. Commun. Netw..

[10] Yin Y., Chen L., Xu Y., Wan J., Zhang H., Mai Z. (2019). QoS prediction for service recommendation with deep feature learning in edge computing environment. Mob. Netw. Appl..

[11] Xie K., Wang X., Liu X., Wen J., Cao J. (2016). Interference-Aware Cooperative Communication in Multi-radio Multi-channel Wireless Networks. IEEE Trans. Comput..

[12] Xie K., Wang X., Wen J., Cao J. (2016). Cooperative Routing with Relay Assignment in Multi-radio Multihop Wireless Networks. IEEE/ACM Trans. Netw. (TON).

[13] Xie K., Ning X., Wang X., He S., Ning Z., Liu X., Wen J., Qin Z. (2017). An efficient privacy-preserving compressive data gathering scheme in WSNs. Inf. Sci..

[14] Xie K., Ning X., Wang X., Xie D., Cao J., Xie G., Wen J. (2017). Recover corrupted data in sensor networks: A matrix completion solution. IEEE Trans. Mob. Comput..

[15] Pan J.S., Kong L., Sung T.W., Tsai P.W., Snášel V. (2018). A clustering scheme for wireless sensor networks based on genetic algorithm and dominating set. J. Internet Technol..

[16] Guo S., Shi Y., Yang Y., Xiao B. (2017). Energy Efficiency Maximization in Mobile Wireless Energy Harvesting Sensor Networks. IEEE Trans. Mob. Comput..

[17] He S., Xie K., Chen W., Zhang D., Wen J. (2018). Energy-aware Routing for SWIPT in Multi-hop Energy-constrained Wireless Network. IEEE Access.

[18] He S., Xie K., Xie K., Xu C., Jin W. (2019). Interference-aware Multi-source Transmission in Multi-radio and Multi-channel Wireless Network. IEEE Syst. J..

[19] Li C., Liu P., Zou C., Sun F., Cioffi J.M., Yang L. (2015). Spectral-efficient cellular communications with coexistent one-and two-hop transmissions. IEEE Trans. Veh. Technol..

[20] Li C., Sun F., Cioffi J.M., Yang L. (2014). Energy efficient MIMO relay transmissions via joint power allocations. IEEE Trans. Circuits Syst. II Express Briefs.

[21] Tang Q., Yang K., Wang J., Luo Y., Li K., Yu F. (2019). Wireless Sensor Network MCDS Construction Algorithms with Energy Consideration for Extreme Environments Healthcare. IEEE Access.

[22] Cao D., Zheng B., Ji B., Lei Z., Feng C. (2018). A robust distance-based relay selection for message dissemination in vehicular network. Wirel. Netw..

[23] He S., Zhang D., Xie K., Qiao H., Zhang J. (2012). Distributed low-complexity channel assignment for opportunistic routing. China Commun..

[24] Zhang J., Jin X., Sun J., Wang J., Sangaih A.K. (2018). Spatial and semantic convolutional features for robust visual object tracking. Multimed. Tools Appl..

[25] Zhang J., Jin X., Sum J., Wang J., Li K. (2019). Dual model learning combined with multiple feature selection for accurate visual tracking. IEEE Access.

[26] Chen Y., Wang J., Chen X., Zhu M., Kai Y., Wang Z., Xia R. (2019). Single-Image Super-Resolution Algorithm Based on Structural Self-Similarity and Deformation Block Features. IEEE Access.

[27] Chen Y., Wang J., Chen X., Sangaiah A.K., Yang K., Cao Z. (2019). Image Super-Resolution Algorithm Based on Dual-Channel Convolutional Neural Networks. Appl. Sci..

[28] He S., Li Z., Tang Y., Liao Z., Wang J., Kim H.J. (2019). Parameters Compressing in Deep Learning. Comput. Mater. Cont..

[29] Liu C., Maso M., Lakshminarayana S., Lee C., Quek T.Q.S. (2015). Simultaneous Wireless Information and Power Transfer under Different CSI Acquisition Schemes. IEEE Trans. Wirel. Commun..

[30] Xiang Z., Tao M. (2012). Robust Beamforming for Wireless Information and Power Transmission. IEEE Wirel. Commun. Lett..

[31] Boshkovska E., Ng D.W.K., Zlatanov N., Schober R. (2015). Practical Non-linear Energy Harvesting Model and Resource Allocation for SWIPT Systems. IEEE Commun. Lett..

[32] Dong Y., Hossain M.J., Cheng J. (2016). Joint Power Control and Time Switching for SWIPT Systems with Heterogeneous QoS Requirements. IEEE Commun. Lett..

[33] Vu Q.D., Tran L.N., Farrell R., Hong E.K. (2015). An Efficiency Maximization Design for SWIPT. IEEE Signal Process. Lett..

[34] Shi Q., Peng C., Xu W., Hong M., Cai Y. (2016). Energy Efficiency Optimization for MISO SWIPT Systems with Zero-Forcing Beamforming. IEEE Trans. Signal Process..

[35] Zong Z., Feng H., Yu F.R., Zhao N., Yang T., Hu B. (2016). Optimal Transceiver Design for SWIPT in K-User MIMO Interference Channels. IEEE Trans. Wirel. Commun..

[36] Jiang R., Xiong K., Fan P., Zhang Y., Zhong Z. (2017). Optimal Design of SWIPT Systems with Multiple Heterogeneous Users Under Non-linear Energy Harvesting Model. IEEE Access.

[37] Qin C., Ni W., Tian H., Liu R.P. (2017). Joint Rate Maximization of Downlink and Uplink in Multiuser MIMO SWIPT Systems. IEEE Access.

[38] Liu Y., Wang X. (2016). Information and Energy Cooperation in OFDM Relaying: Protocols and Optimization. IEEE Trans. Veh. Technol..

[39] Huang G., Zhang Q., Qin J. (2015). Joint Time Switching and Power Allocation for Multicarrier Decode-and- Forward Relay Networks with SWIPT. IEEE Signal Process. Lett..

[40] Diamantoulakis P.D., Ntouni G.D., Pappi K.N., Karagiannidis G.K., Sharif B.S. (2015). Throughput Maximization in Multicarrier Wireless Powered Relaying Networks. IEEE Wirel. Commun. Lett..

[41] Fang B., Zhong W., Jin S., Qian Z., Shao W. (2016). Game-Theoretic Precoding for SWIPT in the DF-based MIMO Relay Networks. IEEE Trans. Veh. Technol..

[42] Zheng G., Ho Z., Jorswieck E., Ottersten B. (2014). Information and Energy Cooperation in Cognitive Radio Networks. IEEE Trans. Signal Process..

[43] Liu P., Gazor S., Kim I.M., Kim D.I. (2015). Energy Harvesting Noncoherent Cooperative Communications. IEEE Trans. Wirel. Commun..

[44] Chu Z., Johnston M., Goff S.L. (2015). SWIPT for wireless cooperative networks. Electron. Lett..

[45] Mahama S., Asiedu D.K.P., Lee K.J. (2017). Simultaneous Wireless Information and Power Transfer for Cooperative Relay Networks with Battery. IEEE Access.

[46] Ren J., Xu M., Chen W., Ding Z., Wang Z. (2017). Coalition Formation Approaches for Cooperative Networks with SWIPT. IEEE Access.

[47] Wang J., Gao Y., Liu W., Sangaiahand A.K., Kim H.J. (2019). Energy Efficient Routing Algorithm with Mobile Sink Support for Wireless Sensor Networks. Sensors.

[48] Wang J., Gao Y., Liu W., Wu W., Lim S.J. (2019). An Asynchronous Clustering and Mobile Data Gathering Schema based on Timer Mechanism in Wireless Sensor Networks. Comput. Mater. Cont..

[49] Li C., Zhang S., Liu P., Sun F., Cioffi J.M., Yang L. (2015). Overhearing protocol design exploiting intercell interference in cooperative green networks. IEEE Trans. Veh. Technol..

[50] Li C., Yang H.J., Sun F., Cioffi J.M., Yang L. (2015). Multiuser overhearing for cooperative two-way multiantenna relays. IEEE Trans. Veh. Technol..

[51] He S., Zhang D., Xie K., Qiao H., Zhang J. (2014). Channel Aware Opportunistic Routing in Multi-Radio Multi-Channel Wireless Mesh Networks. J. Comput. Sci. Technol..

[52] Yu H., Zhang Y., Guo S., Yang Y., Ji L. (2017). Energy efficiency maximization for WSNs with simultaneous wireless information and power transfer. Sensors.

[53] Makare J. (2011). Wireless Resonant Energy Link (Wrel) Demo. http://brightcove.vo.llnwd.net/pd16/media/740838651001/740838651001_1127592260001_R-ID-David-Meyer-V1.mp4.

